# Detección molecular de *Toxoplasma gondii* en carnes para consumo humano en Ibagué, Colombia

**DOI:** 10.7705/biomedica.6251

**Published:** 2022-03-01

**Authors:** Juan David Medina, Laura Alejandra Osorio, Daniel Zabala, Ricardo Wagner de Almeida Vitor, Jorge Enrique Gómez, Julio César Carranza, Gustavo Adolfo Vallejo

**Affiliations:** 1 Laboratorio de Investigaciones en Parasitología Tropical, Universidad del Tolima, Ibagué, Colombia Universidad del Tolima Universidad del Tolima Ibagué Colombia; 2 Departamento de Parasitología, Instituto de Ciencias Biológicas, Universidad Federal de Minas Gerais, Belo Horizonte, Brasil Universidade Federal de Minas Gerais Universidad Federal de Minas Gerais Belo Horizonte Brazil; 3 Grupo de Investigación en Parasitología Molecular (GEPAMOL), Centro de Investigaciones Biomédicas, Universidad del Quindío, Armenia, Colombia Universidad del Quindío , Universidad del Quindío Armenia Colombia

**Keywords:** Toxoplasma gondii, toxoplasmosis, carne, reacción en cadena de la polimerasa, prevalencia, Toxoplasma gondii, toxoplasmosis, meat, polymerase chain reaction, prevalence

## Abstract

**Introducción.:**

*Toxoplasma gondii* es un parásito con gran potencial zoonótico que puede infectar un amplio rango de huéspedes de sangre caliente, incluidos los animales del sector pecuario, lo que causa pérdidas a la industria. En el humano, es patógeno en personas inmunosuprimidas y afecta el desarrollo del feto en infecciones congénitas. Además, se asocia con diversos trastornos del comportamiento en personas sanas. El humano puede adquirir *T. gondii* al consumir carnes contaminadas mal cocidas.

**Objetivo.:**

Determinar la positividad de *T. gondii* en carnes de consumo humano (res, pollo y cerdo) en Ibagué, Colombia.

**Materiales y métodos.:**

Se utilizó la PCR convencional anidada y la secuencia del gen B1 de *T. gondii* como blanco de amplificación. Se tomaron 186 muestras de carne comercializada en la zona urbana de Ibagué (62 de res, 62 de pollo y 62 de cerdo) y se obtuvo el porcentaje de positividad en cada tipo de carne evaluada.

**Resultados.:**

Se encontró un porcentaje de positividad de 18,8 % en las muestras, siendo la carne de cerdo la del mayor porcentaje (22,5 %; 14/62), seguida por las muestras de carne de res (19,3 %; 12/62) y de pollo (14,5 %; 9/62). Los mejores productos amplificados fueron secuenciados en Macrogen, y alineados con las secuencias del gen *B1* depositadas en el GenBank, con lo que se confirmó su identidad.

**Conclusiones.:**

Este es el primer estudio sobre prevalencia de *T. gondii* en carnes para consumo humano en Ibagué y el departamento del Tolima. Se demostró que los tres tipos de carne representan un riesgo para la infección en humanos a nivel local.

## Abstract (en)

**Introduction:**

*Toxoplasma gondii* is a parasite with great zoonotic potential. It can infect a broad range of warm-blooded hosts (including livestock) and causes significant losses in the industry. In humans, it has been described as a pathogen in immunosuppressed people, it affects the fetus development in congenital infections, and is associated with various behavioral disorders in healthy people. Humans can acquire *T. gondii* by consuming undercooked, contaminated meat.

**Objective:**

To determine *T. gondii* positivity (currently unknown) in meat for human consumption (i.e., beef, chicken, and pork) in the city of Ibague, Colombia.

**Materials and methods:**

We used conventional nested PCR and the *T. gondii* B1 gene sequence as amplification target. We collected samples of meat (N=186) sold in the urban area of Ibagué (62 beef, 62 chicken, and 62 pork samples) and determined the *T. gondii* positivity percentage for each type of meat.

**Results:**

The study found an average of 18.8% positivity for all meat samples, pork having the highest percentage (22.5%; 14/62), followed by beef (19.3%; 12/62) and chicken (14.5%; 9/62). The best-amplified products were sequenced by macrogen and aligned with the *B1* gene sequences in GenBank, thereby confirming their identity.

**Conclusions:**

This is the first study of *T. gondii* prevalence in meat for human consumption carried out in the city of Ibagué and the department of Tolima. All three types of meat sampled represent a risk for local human infection.

*Toxoplasma gondii* es un protozoario intracelular obligado de distribución mundial perteneciente al filo Apicomplexa ^1^. Fue descrito por primera vez en 1908 por Nicolle y Manceaux, quienes lo encontraron cuando trabajaban en el norte de África, y por Splendore en Brasil ^2^^,^^3^. Presenta una amplia distribución geográfica y los felinos tienen un papel importante en el ciclo de vida y la epidemiología de la toxoplasmosis, ya que son los huéspedes definitivos que expulsan en sus heces ooquistes resistentes al ambiente ^4^^-^^7^. La principal vía de infección para los animales de producción es la ingestión de alimentos o agua contaminados con ooquistes esporulados liberados en las heces de felinos infectados ^8^^-^^11^.

El humano se puede infectar de la misma forma que los animales de producción, a lo que se suma la manipulación de las cajas de arena para gatos sin la adecuada precaución y la contaminación de las áreas de los parques infantiles con heces de gatos y, por lo tanto, con ooquistes de *T. gondii*, los cuales pueden ser ingeridos de manera accidental ^7^^,^^12^. Lo mismo puede ocurrir por el consumo de carne que contenga quistes tisulares y esté mal cocida ^11^^,^^13^^,^^14^, y por el de frutas y verduras contaminadas durante su manejo ^15^^,^^16^ o su preparación con carne cruda y presentación de contaminación cruzada (17). Se ha demostrado que más del 60 % de algunas poblaciones humanas se han infectado con este parásito, sobre todo en las zonas del mundo de menor altitud, de climas cálidos y húmedos ^6^^,^^18^^-^^20^.

El parásito produce la zoonosis más ampliamente distribuida a nivel mundial, la cual produce síntomas clínicos leves e inespecíficos en la mayoría de los infectados, humanos y animales, o es asintomática ^6^^,^^10^^,^^19^^,^^21^. Se ha descrito que afecta la visión hasta en el 10 % de las personas infectadas ^22^^,^^23^, así como su alta morbilidad en fetos de madres infectadas durante la gestación y su incidencia en lesiones cerebrales en pacientes inmunosuprimidos ^24^^-^^28^.

Aunque la infección crónica solo se desarrolla en un pequeño grupo de personas, en la mayoría se mantiene en estado latente sin aparente riesgo directo para la salud física, aunque se ha asociado al aumento en las tasas de trastornos del comportamiento, incluido el homicidio, el suicidio y otras formas de autoagresión física ^29^^,^^30^, la esquizofrenia y otras enfermedades neuropsiquiátricas ^31^^,^^32^. Además, la infección ocasiona grandes pérdidas económicas y productivas en el sector agropecuario, ya que puede ocasionar aborto espontáneo, retención placentaria, mortinatos o recién nacidos débiles y demacrados en ovejas, cabras y cerdos ^8^^,^^33^^-^^36^.

A nivel mundial, los rangos de prevalencia de *T. gondii* varían ampliamente dependiendo de la muestra, las condiciones biosanitarias, los hábitos o costumbres de la zona y la metodología de detección empleada, entre otros ^9^^,^^37^^,^^38^. Pérez-Grisales, *et al*. ^6^, sugieren que la distribución de *T. gondii* no está restringida a ninguna ecorregión de Colombia; por ejemplo, en el estudio de Triviño-Valencia, *et al*. ^39^, se reportó una prevalencia del 58,6 % del parásito en fuentes de aguas de Armenia; en Sincelejo, se detectó *Toxoplasma* en el 32 % de las carnes analizadas ^40^, y en plantas de sacrificio de Bogotá, una prevalencia del 43 %: 39 % en cerdo, 35 % en res y 29,7 % en pollo ^41^.

Dado que una de las principales formas en que las personas adquieren *T. gondii* es al ingerir carnes mal cocidas, el presente trabajo tuvo como objetivo estimar la prevalencia en carnes destinadas al consumo humano en Ibagué (Tolima, Colombia) como aporte al conocimiento de la epidemiología de la toxoplasmosis a nivel regional y nacional.

## Materiales y métodos

### Área de estudio y tamaño de la muestra

Ibagué es la capital del departamento del Tolima, departamento ubicado en la parte central del país. Se encuentra a 1.285 metros sobre el nivel del mar y su temperatura media es de 21 °C; el municipio tiene un área de 1.450,61 km2 y está conformado por 13 comunas y 445 barrios, y 17 corregimientos y 133 veredas según el Plan de Ordenamiento Territorial (POT) ^42^.

La muestra se calculó utilizando el programa Epi-Info (CDC, versión 7.2.0.1) a partir de los datos recomendados en estudios realizados en otras regiones de Colombia ^40^^,^^41^^,^^43^. Se aplicó un margen de error del 5 % y un nivel de confianza del 95 %, para una proporción de la población esperada del 40 %, en tanto que el efecto del diseño muestral se estimó en 0,5 para un tamaño de población infinita, lo que arrojó 186 muestras de carne distribuidas en 62 de res, 62 de pollo y 62 de cerdo.

Las muestras se obtuvieron de comercializadoras de carne (tiendas de barrio, plazas o almacenes de cadena). Se recolectaron aproximadamente 100 g de cada tejido, los cuales se almacenaron refrigerados a 4 °C hasta su posterior análisis en el laboratorio ^40^^,^^44^.

### Obtención del ADN genómico

A partir de 100 g de carne, se hicieron cortes al azar con una hoja de bisturí estéril hasta obtener 5 g; para la digestión, se empleó solución tampón de lisis (Tris pH 8,5, 100 mM; NaCl 100 mM; EDTA50 mM; SDS 1 %) y proteinasa K (solución de trabajo: 200 μg/ml) a 56 °C durante una hora. La extracción y purificación del ADN a partir de la muestra digerida se efectuó con el método de mezcla de fenol, cloroformo y alcohol isoamílico propuesto por Sambrook, *et al*., y la digestión con ARNasa en una concentración de 50 μg/ml ^45^.

### Amplificación del gen B1 por PCR anidada

Para la detección se empleó una PCR anidada (n-PCR), con el fin de aumentar la especificidad y la sensibilidad, la cual consistió en dos rondas con dos pares de los cebadores empleados por Jones, *et al*. ^46^, los cuales son un poco más cortos que los del estudio original del gen *B1* llevado a cabo por Burg, *et al*. ^47^.

La mezcla de amplificación para la primera reacción consistió en 2 μΙ de solución tampón 10X: 1,2 μl de MgCl_2_ 1,5 mM, 1,6 μl de dNTP, 2,5 mM, 0,8 μl de cebador F, 1 μΜ; 0,8 μl de cebador R, 1 μΜ; 1 μl de Taq polimerasa; 2 μl de muestra de ADN, y agua de tipo 1 hasta completar 20 μl de mezcla de reacción.

La primera ronda de PCR consistió en la amplificación de un fragmento de 127 pb con los cebadores F 5’-GGAACTGCATCCGTTCATGAG-3’ y R 5’-TCTTTAAAGCGTTCGTGGTC-3’, desnaturalización inicial a 94 °C durante cinco minutos, 40 ciclos de desnaturalización a 94 °C durante un minuto, anillado a 53 °C durante un minuto, una extensión a 72 °C durante un minuto y, por último, una extensión a 72 °C durante diez minutos.

La segunda mezcla de amplificación se hizo con las mismas concentraciones de la primera reacción, pero en esta ocasión la muestra consistió en 2 μl del producto de amplificación de la primera ronda. La segunda ronda de PCR consistió en la amplificación de un fragmento de 97 pb con los cebadores F 5’-TGCATAGGTTGCCAGTCACTG-3’ y R 5’-GGCGACCAATCTGCGAATACA-3’, usando como plantilla el producto de la primera amplificación.

Se llevó a cabo una desnaturalización inicial a 94 °C durante cinco minutos, seguida de 30 ciclos de desnaturalización a 94 °C durante un minuto, anillado a 53 °C durante 30 s y una extensión a 72 °C durante 30 s y otra final a 72 °C durante diez minutos. Con el fin de evitar falsos negativos, se empleó como control positivo ADN de la cepa RH de *T. gondii* y, como control negativo, agua de grado Milli-Q estéril ^43^^,^^46^^,^^48^.

### Visualización de los productos

Los productos de la segunda reacción de PCR fueron separados por medio de electroforesis en gel de acrilamida al 6 % en una solución tampón al 1X de tris, ácido bórico y EDTA (TBE) a 80 voltios durante aproximadamente 40 minutos. La tinción se hizo con nitrato de plata y, posteriormente, se tomó la imagen de gel para el análisis. En cada electroforesis se utilizaron un control positivo, uno negativo y un marcador de peso molecular de 1Kb plus DNA Ladder™ (Invitrogen), con el fin de determinar el tamaño de los fragmentos amplificados.

### Secuenciación de los productos e identificación

Los productos que resultaron positivos fueron enviados a secuenciación usando el servicio automático normal de Macrogen™ (Corea), bajo las condiciones de un termociclador BigDye™ en el secuenciador de ADN 3730XL y utilizando los mismos cebadores de amplificación. Las secuencias fueron editadas y alineadas con secuencias de referencia del gen B1 (AF179871.1 y KX270388.1), usando Chromas 2.6.6 y BioEdit 7.2.5. ^49^.

## Análisis estadístico

Los resultados obtenidos se almacenaron en una base de datos de Microsoft Excel y se determinó la frecuencia de infección de cada uno de los tipos de carne, estableciendo la prevalencia y los intervalos de confianza con el programa estadístico InfoStat, versión 2016e. Dado que los datos evaluados fueron de tipo categórico (positivo o negativo), se empleó una prueba de estimación paramétrica basada en una proporción.

## Resultados

Positividad para Toxoplasma gondii en las muestras de carne

**Figura 1:**
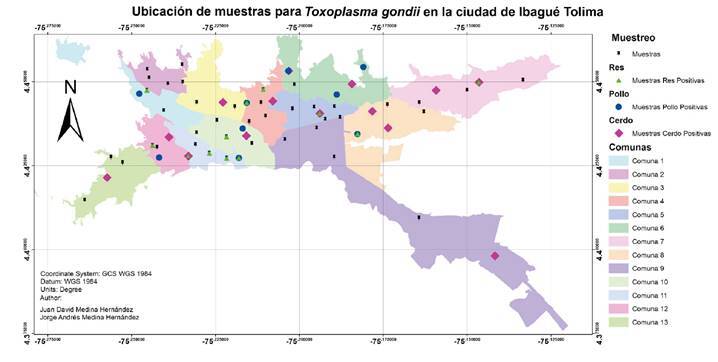
Ubicación de las muestras tomadas en la ciudad de Ibagué

Los resultados obtenidos para cada tipo de carne por barrio se muestran en el cuadro suplementario 1. Con ayuda del programa ArcGIS (versión 10.5), se ubicó en un mapa de la ciudad de Ibagué el total de las muestras con los resultados obtenidos (figura 1). Los cálculos de prevalencia (estimación de proporción) para cada tipo de carne y los intervalos de confianza se presentan en el cuadro 1.

**Cuadro 1:** Estimaciones e intervalos de confianza de las muestras evaluadas

Tipo de carne	Número de muestras positivas	Total de muestras examinadas	Estimación	Intervalos de confanza (95 %)	
				Límite inferior	Límite superior
Res	12	62	0,19	0,1042	0,3137
Pollo	12	62	0,14	0,0686	0,2578
Cerdo	14	62	0,22	0,1293	0,3497
Total	35	186	0,18	0,1320	0,2443

**Figura 2:**
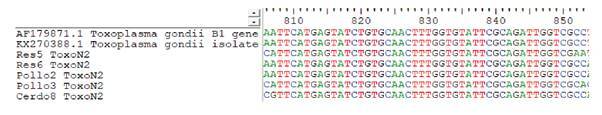
Identidad de las cinco secuencias enviadas a secuenciación, incluidas las de referencia AF179871.1 (2214 pb) y KX270388.1 (803 pb) de *T. gondii* alineadas entre la posición 806 y la 853 de la secuencia de referencia AF179871.1

Se encontró una prevalencia de *T. gondii* del 18,8 % en las muestras de carne (35/186), siendo la prevalencia más alta la de la carne de cerdo (22,5 %) y, la más baja, la de las muestras de carne de pollo (14,5 %) (cuadro 1). Sin embargo, no hubo diferencias significativas entre las prevalencias de las muestras evaluadas. De las 35 muestras positivas, 11 se enviaron a secuenciar debido a que reflejaban una banda fuerte, y cinco de ellas tuvieron una calidad de secuencia aceptable; estas se alinearon (figura 2) y se confirmó que el ADN detectado correspondía a *T. gondii*.

## Discusión

En los estudios a nivel mundial, se reflejan amplios rangos de prevalencia de *T. gondii* en diferentes animales (salvajes, criados para el consumo humano o mascotas) ^6^^,^^50^^-^^55^, los cuales fluctúan desde 0 % hasta 97 % ^34^^,^^56^^,^^57^. En Colombia, también la prevalencia varía entre los diferentes estudios, incluso en ciudades que se encuentren en una misma zona. En el estudio de Lora-Suárez, *et al*., por ejemplo, se puede observar que en tres ciudades del eje cafetero las prevalencias en el mismo tipo de carnes fluctuaron entre 33 % en Manizales (20 % en res, 0 % en pollo y 80 % en cerdo), 65 % en Pereira (45 % en res, 70 % en pollo y 60 % en cerdo) y 60 % en Armenia (80 % en res, 25 % en pollo y 70 % en cerdo) ^43^.

Los resultados del presente trabajo registraron una prevalencia general del 18,8 %, sin diferencia significativa entre los tres tipos de carne, lo que indica, al igual que en otros estudios, que cualquier tipo de carne de animal de sangre caliente puede considerarse una fuente importante de infección por *T. gondii* y que el riesgo asociado con el tipo de carne (pollo, cordero, cerdo o ternera, etc.) varía entre los países según los hábitos alimentarios locales y la tasa de infección en los animales productores ^9^^,^^37^. Los porcentajes de detección del parásito también pueden variar porque la carga parasitaria es un factor importante para detectar con métodos moleculares los protozoos en muestras de tejido ^38^. En Colombia, se ha encontrado que la carne contaminada puede ser la fuente de infección en el 25 % de los casos de toxoplasmosis durante el embarazo ^58^.

Los animales herbívoros como el ganado bovino tienden a adquirir *T. gondii* al consumir heno, pasturas, forraje o agua contaminada con ooquistes provenientes de heces de gatos ^39^^,^^59^. En el presente estudio, se determinó una prevalencia del 19,35 % en las muestras de res analizadas, nivel que se aproxima al reportado por Lora-Suárez, *et al*. ^43^, en Manizales utilizando una PCR anidada en el gen *B1* (20 % en res). Sin embargo, se han obtenido tasas de infección con amplias diferencias entre ellas, incluso, en plantas de sacrificio de una misma ciudad, como en el estudio de Franco-Hernández, *et al*. ^41^, en el cual se empleó la misma metodología de detección y se encontró 63 % de prevalencia del parásito en carnes de res en la planta de tipo I (procesamiento de más de 480 bovinos y más de 400 cerdos cada 8 h) y un 10 % en carnes de res en la planta de tipo II (procesamiento de más de 320 bovinos y más de 240 cerdos cada 8 h).

En carnes de pollo hay una baja prevalencia de infección por *T. gondii* debido, probablemente, a las prácticas de manejo y condiciones de producción intensiva en que son criados; es decir, confinados en galpones y sin contacto con heces de gatos, como sí lo pueden estar aquellos animales que deambulan abiertamente. Dado que los pollos se alimentan directamente de comederos tubulares y no del suelo, que podría estar infectado con ooquistes, se reduce su posibilidad de contraer la infección, aunque pueden contraerla al tomar agua contaminada ^39^^,^^40^^,^^60^. En el presente estudio, se reportó la presencia de *Toxoplasma* en el 14,52 % de las muestras evaluadas, porcentaje cercano al reportado por Cárdenas-Pérez, *et al*. ^61^, usando inmunofluorescencia indirecta (IFI) en muestras de pollo en el departamento de Caldas (16 %).

Debido a que los pollos que se comercializan actualmente tienen aproximadamente 60 días de vida, su tiempo de exposición es poco, por lo que podría esperarse que este también sea un factor determinante para los bajos niveles de prevalencia ^62^.

Los animales de hábitos omnívoros, como el cerdo, pueden adquirir Toxoplasma de la misma manera que los herbívoros, además de infectarse por el consumo de ratones que llevan quistes tisulares o de insectos con hábitos coprófagos ^59^. La carne de cerdo es una de las más consumidas en la actualidad ^43^, y en la que más se ha aislado el parásito, por lo que sus tejidos se han considerado como la fuente más importante de transmisión de *T. gondii* para el hombre ^63^. En el presente estudio, se encontró una prevalencia del 22,58 % en las muestras evaluadas, porcentaje cercano al reportado en lenguas de cerdo por Hernández-Cortázar, *et al*., en México (23,2 %), usando PCR anidada en la secuencia SAG1 ^57^. Sin embargo, en otros estudios a nivel mundial, se han encontrado prevalencias hasta del 96,6 % en los cerdos ^57^^,^^64^.

Hoy no se dispone de pruebas que puedan determinar la fuente de infección, por lo cual la proporción de la población humana que adquiere la infección por ingestión de ooquistes en el medio ambiente, o por el consumo de carne contaminada, no se conoce ^13^^,^^62^. Es claro que la mejor forma de prevención de la infección por *Toxoplasma* es la concientización y educación de la población con mayor riesgo de desarrollar la enfermedad, como las mujeres embarazadas y los pacientes inmunocomprometidos ^58^^,^^65^.

Se sugiere revisar las prácticas y la adecuada manipulación de alimentos con el fin de evitar la contaminación cruzada en su preparación, ya que se ha detectado Toxoplasma en superficies empleadas para la preparación de alimentos ^17^. En la carne, *T. gondii* puede morir por exposición al calor o frío extremos, pues los quistes tisulares en la carne se eliminan calentando la carne a 67 °C o enfriándola a -13 °C ^66^. Los diferentes métodos de producción de los animales, el tamaño de las granjas, las condiciones sanitarias y de bioseguridad, así como las fuentes de agua, las poblaciones urbanas y el uso de productos concentrados fabricados con materias primas de origen animal o vegetal contaminado, pueden tener relación con la prevalencia del parásito en las distintas especies estudiadas.

En conclusión, el presente estudio demostró la utilidad de la PCR anidada para la detección directa de *T. gondii* en carnes comercializadas para el consumo humano, incluso cuando la carga parasitaria es baja. Este constituye el primer trabajo de detección molecular del parásito en Ibagué.

## Archivos suplementarios

**Cuadro suplementario:** 1. Características de las comunas y coordenadas de las muestras tomadas

Compra			Muestra de		
Fecha	Coordenadas	Barrio	Comuna	Res	Pollo	Cerdo
5/05/2017	4.447373 -75.245411	La Pola	1	+	-	-
7/07/2017	4.434714 -75.230658	San Pedro Alejandrino	1	-	-	-
11/09/2017	4.446410 -75.247684	Libertador	1	-	+	-
10/12/2017	4.441307 -75.240402	Centro	1	-	-	-
6/06/2017	4.4493334 -75.239221	Belencito	2	-	-	-
13/08/2017	4.449743 -75.234765	Ancón	2	-	-	-
11/09/2017	4.453609 -75.245356	Santa Bárbara	2	-	-	-
3/12/2017	4.451084 -75.244811	Belén	2	-	-	-
22/01/2018	4.454901 -75.234970	La Aurora	2	-	-	-
24/04/2017	4.443678 -75.215683	Gaitán Parte Alta	3	+	+	-
13/08/2017	4.443840 -75.222876	Antonio Nariño	3	-	-	+
20/10/2017	4.442549 -75.219246	San Simón PB	3	-	-	-
16/12/2017	4.443718 -75.230616	El Carmen	3	-	-	-
5/07/2017	4.439641 -75.210106	Calarcá	4	-	-	-
22/08/2017	4.443640 -75.211440	Gaitán Parte Baja	4	-	-	-
20/10/2017	4.444200 -75.208005	Onzaga	4	-	-	+
12/12/2017	4.438040 -75.214959	Restrepo	4	-	-	-
31/01/2018	4.447586 -75.210789	Sorrento	4	+	-	-
7/06/2017	4.440534 -75.193978	Jordán 8a Etapa	5	+	-	+
28/08/2017	4.442511 -75.189608	La Campiña	5	-	-	-
29/10/2017	4.441789 -75.202082	Jordán 9a Etapa	5	-	-	-
21/12/2017	4.438694 -75.192310	Arrayanes	5	-	-	-
31/01/2018	4.442365 -75.195641	Jordán 7-,	5	-	-	-
19/07/2017	4.454375 -75.180932	La Gaviota	6	-	+	-
9/09/2017	4.446146 -75.188868	Entre Ríos	6	-	+	-
2/11/2017	4.449237 -75.184412	Cañaveral	6	-	-	+
16/12/2017	4.448963 -75.201540	Plazas del Bosque	6	-	-	-
11/02/2018	4.453173 -75.203239	Los Mandarinos	6	-	+	+
7/06/2017	4.449820 -75.146532	Salado	7	+-		+
15/09/2017	4.447365 -75.159341	San Pablo	7	-	-	+
2/11/2017	4.443637 -75.164304	Santa Ana	7	-	-	-
16/01/2018	4.447369 -75.150075	Urb. Portales del Norte	7	-	-	-
4/02/2018	4.4503 -75.133391	Modelia II	7	-	-	-
2/08/2017	4.44094 -75.162977	Topacio	8	-	-	-
9/08/2017	4.443090 -75.173774	Caminos del Norte/ Plaza del Jardín	8	-	-	-
17/11/2017	4.434414 -75.182698	Ciudadela Simón Bolívar	8	+	+	-
21/12/2017	4.441160 -75.178343	El Bunde	8	-	-	+
4/02/2018	4.436170 -75.173660	Ciudadela Simón Bolívar II	8	-	-	+
6/07/2017	4.439268 -75.187921	Valparaíso	9	-	-	-
28/08/2017	4.436080 -75.194837	Jordán 3a Etapa	9	-	-	-
27/10/2017	4.398124 -75.141801	Picaleña	9	-	-	+
3/12/2017	4.432674 -75.204332	Hacienda Piedra Pintada	9	-	-	-
23/01/2018	4.409308 -75.164390	Santa Rita	9	-	-	-
4/02/2018	4.427452 -75.189630	Villa Café	9	-	-	-
26/04/2017	4.433871 -75.215873	Las Palmas	10	-	-	+
9/09/2017	4.436033 -75.216881	Montealegre	10	-	+	-
15/09/2017	4.433538 -75.221728	La Francia	10	+	-	-
13/10/2017	4.431488 -75.214161	Santa Helena	10	-	-	-
16/01/2018	4.438397 -75.224590	Hipódromo	10	-	-	-
16/07/2017	4.431223 -75.230947	12 de Octubre	11	-	-	-
1/08/2017	4.427266 -75.218032	Los Mártires	11	+	+	-
17/11/2017	4.427819 -75.233129	Refugio	11	+	-	+
14/01/2018	4.428634 -75.226832	Las Brisas	11	+	-	-
4/02/2018	4.42713 -75.221676	Bosque	11	+	-	-
21/07/2017	4.430935 -75.243748	Ricaurte	12	+	-	-
9/08/2017	4.430415 -75.242418	Galán	12	-	-	-
6/10/2017	4.427426 -75.241778	Kennedy	12	-	+	-
26/11/2017	4.433504 -75.238963	Yuldaima	12	-	-	+
21/07/2017	4.421427 -75.257337	Granada	13	-	-	+
21/07/2017	4.425848 -75.252700	Miramar	13	-	-	-
26/11/2017	4.414616 -75.263979	Boquerón	13	-	-	-
14/01/2018	4.427462 -75.256117	San Isidro	13	-	-	-

## References

[1] Ajzenberg D, Dumètre A, Dardé ML (2005). Multiplex PCR for typing strains of Toxoplasma gondii. J Clin Microbiol.

[2] Tenter AM, Heckeroth AR, Weiss LM (2000). Toxoplasma gondii: From animals to humans. Int J Parasitol.

[3] Weiss LM, Dubey JP (2009). Toxoplasmosis: A history of clinical observations. Int J Parasitol.

[4] Dubey JP, Navarro IT, Sreekumar C, Dahl E, Freire RL, Kawabata HH (2004). Toxoplasma gondii infections in cats from Parana, Brazil: Seroprevalece, tissue distribution, and biologic and genetic characterization of isolates. J Parasitol.

[5] Yang Y, Ying Y, Verma SK, Cassinelli ABM, Kwok OCH, Liang H (2015). Isolation and genetic characterization of viable Toxoplasma gondii from tissues and feces of cats from the central region of China. Vet Parasitol.

[6] Pérez-Grisales LJ, Cruz-Moncada M, Peláez-Sánchez R, Díaz-Nieto JF (2021). Toxoplasma gondii infection in Colombia with a review of hosts and their ecogeographic distribution. Zoonoses Public Health.

[7] Simon JA, Kurdzielewicz S, Jeanniot E, Dupuis E, Marnef F, Aubert D (2017). Spatial distribution of soil contaminated with Toxoplasma gondii oocysts in relation to the distribution and use of domestic cat defecation sites on dairy farms. Int J Parasitol.

[8] Maharana B, Panigrahi M, Baithalu RK, Parida S, Allaie IM (2010). Toxoplasmosis: Beware of cats !!!. Vet World.

[9] Guo M, Lambertini E, Buchanan RL, Dubey JP, Hill DE, Gamble HR (2017). Quantifying the risk of human Toxoplasma gondii infection due to consumption of fresh pork in the United States. Food Control.

[10] Ramos-Valencia OA, Vásquez-Arteaga LR (2017). Toxoplasmosis humana, variables y determinantes sociales en un municipio rural en Colombia. Red Científica Iberoamericana.

[11] Cruz-Quevedo M, Hernández-Cruz A, Dorta-Contreras AJ (2019). nexo entre biología, respuesta inmune y clínica en la infección por Toxoplasma gondii. Rev Cuba Investig Biomédicas.

[12] Yekkour F, Aubert D, Mercier A, Murat J-B, Khames M, Nguewa P (2017). First genetic characterization of Toxoplasma gondii in stray cats from Algeria. Vet Parasitol.

[13] Dubey JP, Rajendran C, Ferreira LR, Martins J, Kwok OCH, Hill DE (2011). High prevalence and genotypes of Toxoplasma gondii isolated from goats, from a retail meat store, destined for human consumption in the USA. Int J Parasitol.

[14] Zulpo DL, Headley SA, Biazzono L, da Cunha IAL, Igarashi M, de Barros LD (2012). Oocyst shedding in cats vaccinated by the nasal and rectal routes with crude rhoptry proteins of Toxoplasma gondii. Exp Parasitol.

[15] Cunha MM, Carneiro ACA V, Costa JGL, Vitor RWA (2016). Genotyping of Toxoplasma gondii directly from human and animal biological samples: From partial genotypes to a new genotype. J Parasitol.

[16] Lass A, Pietkiewicz H, Szostakowska B, Myjak P (2012). The first detection of Toxoplasma gondii DNA in environmental fruits and vegetables samples. Eur J Clin Microbiol Infect Dis.

[17] Luna JC, Zamora A, Hernández-Arango N, Muñoz-Sánchez D, Pinzón MI, Cortés-Vecino JA (2019). Food safety assessment and risk for toxoplasmosis in school restaurants in Armenia, Colombia. Parasitol Res.

[18] Centers for Disease Control and Prevention, CDC Toxoplasmosis - Epidemiology & Risk Factors. 2018.

[19] Giraldo-Ospina B, Garzón-Castaño SC, López-Muñoz DF, Cardozo-Ríos L, Millán-Benavidez N (2019). Seroprevalencia de anticuerpos anti-Toxoplasma gondii en mujeres de 18 años de una localidad de Colombia. Ginecol Obs Mex.

[20] Ramírez AM, Ríos YK, Galvis NF, Entrena E, Mario NV, Rangel DM (2019). Seroprevalencia y detección molecular de Toxoplasma gondii en donantes de un banco de sangre de Cúcuta, Colombia. Biomédica.

[21] Restrepo-Isaza M (2007). Toxoplasmosis: Parasitic zoonosis. CES Med.

[22] Gómez-Marín JE, Zuluaga JD, Pechené-Campo EJ, Triviño J, de la Torre A (2018). Polymerase chain reaction (PCR) in ocular and ganglionar toxoplasmosis and the effect of therapeutics for prevention of ocular involvement in South American setting. Acta Trop.

[23] de la Torre A, Valdés-Camacho J, de Mesa CL, Uauy-Nazal A, Zuluaga JD, Ramírez-Páez LM (2019). Coinfections and differential diagnosis in immunocompetent patients with uveitis of infectious origin. BMC Infect Dis.

[24] Luft BJ, Hafner R, Korzun AH, Leport C, Antoniskis D, Bosler EM (1993). Toxoplasmic encephalitis in patients with the acquired immunodeficiency syndrome. N Engl J Med.

[25] Montoya J, Liesenfeld O (2004). Toxoplasmosis. Lancet.

[26] Barrios JE, Durán-Botello C, González-Velásquez T (2012). Nephrotic syndrome with a nephritic component associated with toxoplasmosis in an immunocompetent young man. Colombia Médica.

[27] Cortés AD, Aguirre N (2018). Toxoplasmosis aguda diseminada fatal en una paciente adulta inmunocompetente proveniente del Pacífico colombiano. Biomédica.

[28] Enriquez-Marulanda A, Valderrama-Chaparro J, Parrado L, Vélez JD, Granados AM, Orozco JL (2017). Cerebral toxoplasmosis in an MS patient receiving Fingolimod. Mult Scler Relat Disord.

[29] Pedersen MG, Mortensen PB, Norgaard-Pedersen B, Postolache TT (2012). Toxoplasma gondii infection and self-directed violence in mothers. Arch Gen Psychiatry.

[30] Lester D (2012). Toxoplasma gondii and homicide. Psychol Rep.

[31] Fabiani S, Pinto B, Bruschi F (2013). Toxoplasmosis and neuropsychiatric diseases: Can serological studies establish a clear relationship?. Neurol Sci.

[32] Torres L, Robinson S, Kim D, Yan A, Cleland TA, Bynoe MS (2018). Toxoplasma gondii alters NMDAR signaling and induces signs of Alzheimer’s disease in wild-type, C57BL/6 mice. J Neuroinflammation.

[33] Camossi LG, Greca-Júnior H, Correa APFL, Richini-Pereira VB, Silva RC, Da Silva A V. (2011). Detection of Toxoplasma gondii DNA in the milk of naturally infected ewes. Vet Parasitol.

[34] Ferra B, Holec-Gasior L, Kur J (2015). Serodiagnosis of Toxoplasma gondii infection in farm animals (horses, swine, and sheep) by enzyme-linked immunosorbent assay using chimeric antigens. Parasitol Int.

[35] Meireles LR, Galisteo AJ, Pompeu E, Andrade HF (2004). oxoplasma gondii spreading in an urban area evaluated by seroprevalence in free-living cats and dogs. Trop Med Int Heal.

[36] Fernández-Escobar M, Calero-Bernal R, Benavides J, Regidor-Cerrillo J, Guerrero-Molina MC, Gutiérrez-Expósito D (2020). Isolation and genetic characterization of Toxoplasma gondii in Spanish sheep flocks. Parasit Vectors.

[37] Robert-Gangneux F, Dardé ML (2012). Epidemiology of and diagnostic strategies for toxoplasmosis. Clin Microbiol Rev.

[38] Yousefvand A, Mirhosseini SA, Ghorbani M, Mohammadzadeh T, Moghaddam MM, Mohammadyari S (2021). Molecular and serological detection and of Toxoplasma gondii in small ruminants of southwest Iran and the potential risks for consumers. J Consum Prot Food Saf.

[39] Triviño-Valencia J, Lora-Suárez FM, Zuluaga JD, Gómez-Marín JE (2016). Detection by PCR of pathogenic protozoa in raw and drinkable water samples in Colombia. Parasitol Res.

[40] Campo-Portacio DM, Discuviche-Rebolledo MA, Blanco-Tuirán PJ, Montero-Pérez YM, Orozco-Méndez KE, Assia-Mercado YM (2014). Detección de Toxoplasma gondii por amplificación del gen B1 en carnes de consumo humano. Infectio.

[41] Franco-Hernández EN, Acosta A, Cortés-Vecino J, Gómez-Marín JE (2016). Survey for Toxoplasma gondii by PCR detection in meat for human consumption in Colombia. Parasitol Res.

[42] Alcaldía Municipal de Ibagué (2020). Ibagué Vibra - Localización.

[43] Lora-Suárez FM, Aricapa H, Pérez JE, Arias L, Idarraga S, Mier D (2007). Detección de Toxoplasma gondii en carnes de consumo humano por la técnica de reacción en cadena de la polimerasa en tres ciudades del eje cafetero. Infectio.

[44] Boughattas S, Ayari K, Sa T, Aoun K, Bouratbine A (2014). Survey of the parasite Toxoplasma gondii in human consumed ovine meat in Tunis City. PLoS One.

[45] Sambrook J, Fritsch EF, Maniatis T, Ford N, Nolan C, Ferguson M (1989). Molecular cloning: A laboratory manual.

[46] Jones CD, Okhravi N, Adamson P, Tasker S, Lightman S (2000). Comparison of PCR detection methods for B1, P30 and 18S rDNA genes of T. gondii in aqueous humor. Invest Ophthalmol.

[47] Burg JL, Grover CM, Pouletty P, Boothroyd JC (1989). Direct and sensitive detection of a pathogenic protozoan, Toxoplasma gondii, by polymerase chain reaction. J Clin Microbiol.

[48] Ponce NC, Gómez-Marín JE (2003). Estandarización y validación clínica de la prueba de reacción en cadena de la polimerasa ( PCR ) para diagnóstico de toxoplasmosis cerebral en pacientes infectados por el VIH. Infectio.

[49] Hall TA (1999). BioEdit a user-friendly biological sequence alignment editor and analysis program for Windows 95,98, NT. Nucleic Acids Symp Ser.

[50] Hernández-Cortázar IB, Acosta-Viana KY, Guzmán-Marín E, Segura-Correa JC, Ortega- Pacheco A, Carrillo-Martínez JR (2016). Toxoplasma gondii in women with recent abortion from Southern México. Asian Pacific J Trop Dis.

[51] Coelho C, Vieira-Pinto M, Vilares A, Gargaté MJ, Rodrigues M, Cardoso L (2020). PCR detection of Toxoplasma gondii in European wild rabbit (Oryctolagus cuniculus) from Portugal. Microorganisms.

[52] Navarrete MG, Cordeiro MD, Batista Y, Alonso JC, Márquez M, Roque E (2017). Serological detection of Toxoplasma gondii in domestic dogs in the western region of Cuba. Vet Parasitol Reg Stud Reports.

[53] Cong W, Qin SY, Meng QF, Zou FC, Qian AD, Zhu XQ (2016). Molecular detection and genetic characterization of Toxoplasma gondii infection in sika deer (Cervus nippon) in China. Infect Genet Evol.

[54] Plaza J, Dámek F, Villena I, Innes EA, Katzer F, Hamilton CM (2020). Detection of Toxoplasma gondii in retail meat samples in Scotland. Food Waterborne Parasitol.

[55] Sroka J, Karamon J, Wójcik-Fatla A, Piotrowska W, Dutkiewicz J, Bilska-Zajac E (2020). Toxoplasma gondii infection in slaughtered pigs and cattle in Poland: Seroprevalence, molecular detection and characterization of parasites in meat. Parasit Vectors.

[56] Al-Kappany YM, Rajendran C, Ferreira LR, Kwok OCH, Abu-Elwafa SA, Hilali M (2010). High prevalence of toxoplasmosis in cats from Egypt: Isolation of viable Toxoplasma gondii, tissue distribution, and isolate designation. J Parasitol.

[57] Hernández-Cortázar IB, Acosta-Viana KY, Guzmán-Marín E, Ortega-Pacheco A, Torres- Acosta JF de J, Jiménez-Coello M (2016). Presence of Toxoplasma gondii in pork intended for human consumption in tropical southern México. Foodborne Pathog Dis.

[58] Díaz L, Zambrano B, Chacón G, Rocha A, Díaz S (2010). Toxoplasmosis y embarazo. Rev Obs Ginecol Venez.

[59] Kijlstra A, Jongert E (2008). Control of the risk of human toxoplasmosis transmitted by meat. Int J Parasitol.

[60] Liu XC, He Y, Han DG, Zhang ZC, Li K, Wang S (2017). Detection of Toxoplasma gondii in chicken and soil of chicken farms in Nanjing region, China. Infect Dis Poverty.

[61] Cárdenas-Pérez JE, Aricapa-Giraldo HJ, Ríos-Candelo SM, Guevara-Garay LA, Meza- Osorio JA, Correa-Salgado RA (2006). Prevalencia de anticuerpos anti-Toxoplasma gondii en cuatro especies de consumo humano en Caldas-Colombia. Biosalud.

[62] Guo M, Dubey JP, Hill D, Buchanan RL, Gamble HR, Jones JL (2015). Prevalence and risk factors for Toxoplasma gondii infection in meat animals and meat products destined for human consumption. J Food Prot.

[63] Dubey JP (2009). Toxoplasmosis in pigs -The last 20 years. Vet Parasitol.

[64] Bacci C, Vismarra A, Mangia C, Bonardi S, Bruini I, Genchi M (2015). Detection of Toxoplasma gondii in free-range, organic pigs in Italy using serological and molecular methods. Int J Food Microbiol.

[65] Opsteegh M, Kortbeek TM, Havelaar AH, van der Giessen JWB (2015). Intervention strategies to reduce human Toxoplasma gondii disease burden. Clin Infect Dis.

[66] Hill D, Dubey JP (2002). Toxoplasma gondii: Transmission, diagnosis, and prevention. Clin Microbiol Infect.

